# Reciprocal Relationship Between Self-Control Belief and Gaming Disorder in Children and Adolescents: Longitudinal Survey Study

**DOI:** 10.2196/59441

**Published:** 2025-01-15

**Authors:** Shimin Zhu, Di Qi

**Affiliations:** 1 Department of Applied Social Sciences, The Hong Kong Polytechnic University, Rm GH348, 11 Yuk Choi Road, Hung Hom, Kowloon, Hong Kong, China (Hong Kong), 852 27665787; 2 Mental Health Research Centre, The Hong Kong Polytechnic University, Hong Kong, China (Hong Kong)

**Keywords:** growth mindset, gaming disorder, cross-lagged panel model, children, self-control, adolescents

## Abstract

**Background:**

Children and adolescents are often at the crossroads of leisure gaming and excessive gaming. It is essential to identify the modifiable psychosocial factors influencing gaming disorder development. The lay theories of self-control (ie, the beliefs about whether self-control can be improved, also called self-control mindsets) may interplay with self-control and gaming disorder and serve as a promising influential factor for gaming disorder.

**Objective:**

This study aims to answer the research questions “Does believing one’s self-control is unchangeable predict more severe gaming disorder symptoms later?” and “Does the severity of gaming disorder symptoms prospectively predict self-control mindsets?” with a 1-year, 2-wave, school-based longitudinal survey.

**Methods:**

A total of 3264 students (338 in grades 4‐5 and 2926 in grades 7‐10) from 15 schools in Hong Kong participated in the classroom surveys. We used cross-lagged panel models to examine the direction of the longitudinal association between self-control mindsets and gaming disorder.

**Results:**

A bidirectional relationship was found between self-control mindsets and gaming disorder symptom severity (the cross-lagged path from mindsets to gaming disorder: regression coefficients [*b*] with 95% CI [0.070, 0.020-0.12o, *P*=.006]; and from gaming disorder to mindsets: *b* with 95% CI [0.11, 0.060-0.160, *P*<.001]). Subgroup analyses of boy and girl participants revealed that more growth mindsets regarding self-control predicted less severe gaming disorder symptoms in girls (*b*=0.12, 95% CI 0.053-0.190, *P*=.001) but not in boys (*b*=0.025, 95% CI –0.050 to 0.100, *P*=.51), while more severe gaming disorder symptoms predicted a more fixed mindset of self-control in both boys (*b*=0.15, 95% CI 0.069-0.230, *P*<.001) and girls (*b*=0.098, 95% CI 0.031-0.170, *P*=.004) after 1 year.

**Conclusions:**

Our findings demonstrated the negative impact of gaming disorder on one’s self-control malleability beliefs and implied that promoting a growth mindset regarding self-control might be a promising strategy for gaming disorder prevention and early intervention, especially for girls.

## Introduction

Internet gaming is popular worldwide, especially among young people. Over 87% of internet users aged 16‐24 years around the world play video games [1]. However, underdevelopment of cognitive control may lead to excessive gaming among children and adolescents, rendering them susceptible to gaming disorder [2]. The updated prevalence rate of internet gaming disorder (IGD) in adolescents and young adults worldwide was 9.9% in recent meta-analyses [3], and the prevalence of probable IGD among Hong Kong adolescents was reported to be as high as 15.6% [4]. Excessive gaming greatly increased due to the social distancing and quarantine measures during the COVID-19 pandemic [5,6], and this trend might continue in the postpandemic era as the number of video game users keeps increasing worldwide [7]. The negative consequences of excessive gaming include, but are not limited to, lower academic achievement, reduced mental wellness, and comorbid psychiatric disorders [8,9]. Moreover, gaming disorder is difficult to treat with medication as patients usually refuse it [10]. Thus, there is a pressing need to identify the modifiable psychological factors associated with problematic gaming behavior to develop more effective preventive interventions.

Self-control plays a central role in managing both the amount and timing of game playing [11]. Lacking self-control over gaming is a core feature of gaming disorder in both the *DSM-5* (*Diagnostic and Statistical Manual of Mental Disorders, Fifth Edition*) and *ICD-11* (*International Classification of Diseases, Eleventh Edition*) [12,13]. Plentiful literature has observed the negative association between self-control and gaming disorder [14-16]. Apart from the direct effects, research has also found indirect effects of self-control on gaming disorder via maladaptive gaming-related cognitions [16]. However, few studies have investigated the associations between gaming-related behavior and implicit theories. Implicit theories are referred to as mindsets, are lay theories or unconscious beliefs about the changeability of one’s attributes. If one has a growth mindset (also known as incremental theories), one believes that one’s attributes can be improved and changed; while if one has a fixed mindset (ie, entity theories), one believes that attributes are fixed entities and cannot change. One previous study tested the links between implicit theories of intelligence and serious game learning [17], showing that players with a growth mindset performed better in serious games and demonstrated more self-directed learning during game playing than players with a fixed mindset. However, as mindsets are domain-specific [18], how self-control mindsets interact with gaming behavior and disorder is yet unknown.

Based on the literature, we propose that self-control mindsets may be a promising influential factor for gaming disorder. Previous studies have demonstrated that beliefs about the malleability of human attributes influence self-regulatory processes and outcomes [19,20]. Further meta-analytic studies have revealed that in contrast to fixed mindsets, growth mindsets predicted the 3 core self-regulatory processes of goal setting, goal operating, and goal monitoring, which then predicted goal achievement [21]. A later intervention study indicated that promoting a growth mindset for self-regulation improved self-regulation in both laboratory settings and daily life via decreased effort avoidance and improved persistence [22], corroborating that cultivating a growth mindset might be a promising way to sustain self-control and protect adolescents from excessive gaming and gaming disorder.

In terms of the associations between self-control mindsets and gaming disorder, there are 2 plausible directions. One possibility is that fixed self-control mindsets predict gaming disorder. Adolescents with fixed self-control mindsets may deem themselves as inherently lacking self-control; as a result, it is less likely for them to make continuous efforts to execute self-control to stop their gaming behavior, leading them to play more frequently and for longer periods and increasing the possibility of developing gaming disorder. Besides, addictive behaviors such as gaming disorder may also lead to fixed mindsets about self-control. For instance, teens who are preoccupied with and lack self-control for gaming may be more likely to believe their self-control is fixed and not improvable. Therefore, it is necessary to delineate the reciprocal relationships between self-control mindsets and gaming disorder to contribute to theory development and practical direction. Disentangling the direction of the link between self-control mindsets and gaming disorder can enrich the understanding of developmental trajectories. Furthermore, the 2 alternative prediction models provide different implications for prevention and intervention [23].

Literature has consistently found gaming disorder rates were much higher in boys than in girls [3,24]. Furthermore, previous studies have also demonstrated distinct levels of gaming disorder symptoms among men and women who play games [14], and that girls tend to better understand the reasons for and potential dangers of gaming disorder [25]. Apart from the gender differences in adolescents’ gaming behaviors observed, including boys playing more, presenting more disordered gaming and preferring more of competitive genres than girls, gender was also shown to moderate personality’s association with gaming disorder, thus gender is highly relevant and gender-dependent differences need more attention when studying gaming-related behaviors [26]. Establishing how the relationships between self-control mindsets and gaming disorder differ across genders is key to tailoring intervention plans for girls and boys.

In this study, we used longitudinal data across 2 academic years from primary and secondary schools to identify the reciprocal associations between beliefs about the malleability of self-control and gaming behavior via a cross-lagged panel model (CLPM). This study aimed to answer the research questions “Does believing one’s self-control is unchangeable predict more severe gaming disorder symptoms after one year?” and “Does the severity of gaming disorder symptoms prospectively predict self-control mindsets?” Additionally, we aimed to explore the gender differences in the reciprocal relationships between self-control mindsets and gaming disorder by examining the cross-lagged models among girl and boy participants, respectively.

## Methods

### Procedure and Participants

This was a 2-wave, longitudinal, and school-based survey study. This study crossed 2 academic years, with surveys administered in June 2021 (T1) and June 2022 (T2). We sent invitations to 23 primary and secondary schools across Hong Kong. School invitations stopped when 4 primary schools and 11 secondary schools agreed to participate. Students from grades 4‐6 at the primary schools and students from grades 7‐11 at the secondary schools were invited to participate. The information sheet and parental consent forms detailing research objectives, possible risks, and participation benefits were sent to parents through schools.

The surveys were conducted in classrooms by trained research assistants who provided guidance and answered queries when appropriate. Both English and Chinese versions of the questionnaire were provided. Once completed, the questionnaires were packed, sealed, and returned to the laboratory.

A total of 4286 students (aged 10‐20 years) from 4 primary schools and 11 secondary schools participated in the T1 survey. Students from grades 6 (n=157) and 11 (n=381) who could not be traced at T2 due to graduation were excluded from analyses. As this study was focused on investigating students’ gaming behaviors, we further excluded those reporting they did not play games at both T1 and T2 (n=484). Ultimately, 3264 participants (1539 boys; 338 students in grades 4‐5 and 2926 students in grades 7‐10) were included in the data analyses. The baseline demographic characteristics of the final sample of 3264 students are displayed in Table 1.

**Table 1.** Baseline characteristics of participants (N=3264).

Variable	Value
Age (years), mean (SD)	14.02 (1.64)
**Gender, n (%)**	
Male	1539 (47.2)
Female	1725 (52.8)
**Grade, n (%)**	
Grade 4 (primary)	180 (5.5)
Grade 5 (primary)	158 (4.8)
Grade 7 (secondary)	998 (30.6)
Grade 8 (secondary)	765 (23.4)
Grade 9 (secondary)	634 (19.4)
Grade 10 (secondary)	529 (16.2)
**Ethnicity, n (%)**	
Chinese	3200 (98)
Non-Chinese	64 (2)
**Family structure, n (%)**	
With both parents	2581 (79.1)
With single parent	549 (16.8)
With neither parent	123 (3.8)
Missing	11 (0.3)
**Socioeconomic status, n (%)**	
Low family affluence	25 (0.8)
Medium family affluence	2670 (81.8)
High family affluence	410 (12.6)
Missing	159 (4.9)

Among the total 3264 participants, 2834 participants (1347 boys; 86.8% of the T1 sample) from the 4 primary schools (n=303) and 11 secondary schools (n=2531) remained in the T2 survey while 430 participants were lost to follow up. Attrition analysis was conducted between participants who participated in both time points (n=2834) and only T1 (n=430), and only significant age differences (*P*=.049) were found. Specifically, those who were older were more likely to drop out at T2. Thus, age was added as an auxiliary variable [27] in our cross-lagged analyses later.

### Measures

Gaming disorder symptoms were measured using an adapted version of the Game Addiction Scale (GAS) for Chinese youths consisting of 7 items [28,29]. The GAS was developed, conceptually, based on the diagnostic criteria for IGD in the *DSM-5* [29]. A sample item is “I have thought all day long about playing a game.” Respondents needed to indicate the extent of their endorsement of the 7 symptoms on a 6-point Likert scale (1=strongly disagree, 6=strongly agree). Higher mean GAS scores represented more severe gaming disorder symptoms. Both Cronbach α and McDonald ω were 0.85 at T1 and 0.86 at T2, showing good reliability.

Mindset of self-control or self-control mindset (MS) was measured using an adapted version of the 4-item Entity Beliefs Subscale of the Implicit Theories of Intelligence Scale [30]. The sentences referred to the descriptions in the original scale but included the word “self-control” instead of “intelligence,” such as “People have a certain amount of self-control, and there isn’t much they can do to change it.” The 4 items were rated on a 6-point Likert scale (1=strongly disagree, 6=strongly agree). The mean score indicated respondents’ self-control mindset (ie, the extent to which they believe their self-control is fixed and unchangeable). The higher the mean score, the more fixed the respondents’ self-control mindset was, while a lower mean score indicated more of a growth self-control mindset (ie, belief in changeability of self-control). The reliability of this scale was also adequately high, as both Cronbach α and McDonald ω were 0.85 at T1 and 0.84 at T2.

Self-control was measured by 9 items from the Brief Self-Control Scale [31,32], which assesses individual differences in people’s traits of self-control. Based on the confirmatory factor analysis results of Unger et al [32] for the Chinese version of Tangney’s Self-Control Scale, the 13 items in the Brief Self-Control measure belonged to 4 factors (5 items on general capacity for self-discipline, 2 items on deliberate or nonimpulsive action, 2 items on healthy habits, and 4 items on work ethics). We maintained 1 item that showed the largest loading on the factor of general capacity for self-discipline (“I have a hard time breaking bad habits”) and kept all 8 items on other factors. The items were also measured on a 6-point Likert scale (1=strongly disagree, 6=strongly agree). Some items were reverse-scored, and a higher mean score on all 9 items represented better self-control. This scale’s Cronbach α was 0.80 for T1 and 0.81 for T2, and the McDonald ω was 0.81 for T1 and 0.82 for T2, indicating good reliability as well.

Gaming time (GT) was measured by a single item that asked participants to report their average time (ie, none, half an hour, 1 hour, 2 hours, 3 hours, 4 hours, 5 hours, and more than 5 hours) spent playing video games each day.

Sociodemographic information including gender, age, grade, ethnicity, family structure, and socioeconomic status was collected. Family structure was measured as whether participants lived with both, one, or neither of their parents. The specific details of measuring socioeconomic status and the questionnaires used are reported in Multimedia Appendix 1.

Attention-checking questions were added to the T2 survey to screen out participants not taking the questionnaire seriously. Three items were distributed throughout the questionnaire [33] and instructed respondents to choose one specific option such as choosing “strongly agree” among the 5 choices from 1=strongly disagree to 5=strongly agree.

### Statistical Analysis

All analyses in this study were conducted in SPSS (version 26; IBM Corp) and R (R Foundation), with the CLPM analyses conducted using the *lavaan* package in R. Descriptive statistical analysis was conducted for each variable based on the available data. Independent samples *t* tests (2-tailed) were performed to compare gender differences in the severity of gaming disorder symptoms (GAS) and self-control mindset at baseline. After this, Pearson correlation analyses were used to measure the correlations between all variables. Next, CLPM analysis using structural equation modeling was performed to examine the directions of relationships between GAS and self-control mindset. Measurement invariance across the 2 waves was first tested for all variables except GT (single-item assessment), as a prerequisite for the CLPM analyses. To determine which level of measurement invariance was upheld among the configural, metric, scalar, and residual models, a decrease of the comparative fit index (CFI) of no more than 0.01 [34] was regarded as the more parsimonious model being satisfied. Residual invariance was turned out to be upheld for all variables including GAS, self-control mindset, and self-control. Thus, the scale scores of these constructs can be compared across time.

The CLPM analyses with self-control mindset and GAS as latent variables were then conducted, with scale items serving as indicators for each latent variable (self-control mindset and GAS), which helps partially out measurement errors and test all pathways of research interest at the same time [34]. The maximum likelihood estimation with robust SEs was used for the structural equation modeling [35]. Model fit was evaluated using the following criteria [34]: the CFI (acceptable >0.90 and good >0.95), the root mean square error of approximation (RMSEA; acceptable <0.08 and good <0.05), and the standardized root mean square residual (SRMR; acceptable <0.08 and good <0.05).

As we added attention-checking questions in the T2 assessment to ensure data quality, those not answering all 3 attention-checking questions correctly (n=645) were seen as failing attention-checking, and their T2 data were treated as missing, as the same as those lost to follow up at T2 (n=430). When conducting the CLPM analyses, missing data was handled using full information maximum likelihood procedures [36] with age as an auxiliary variable [27].

To test whether the cross-lagged effects were different between boys and girls, subgroup CLPM analyses with age as the auxiliary variable were conducted for girl and boy participants, respectively. We also conducted CLPM analyses to investigate the directions of the relationships between self-control mindset and GT and between self-control mindset and self-control following the same procedures set out above.

### Ethical Considerations

Ethical approval was obtained from the Human Subjects Ethics Sub-Committee of the Hong Kong Polytechnic University (reference: HSEARS20210414004-01). Parental and respondents’ written consent was obtained before this study. Students were assured that their participation was voluntary, that they could withdraw at any time, and that their responses would not be accessed by teachers or parents. All personally identifiable data were stored on a secure server and are to be destroyed 3 years after the conclusion of this study. We made sure participants’ personal data were not identifiable in our research report. We did not apply any images in this paper or supplementary material that could identify the individual participants. Each participant received a piece of stationery (worth about US $5) after completion of each survey as compensation.

## Results

The mean GAS score of the sample at baseline was 2.85 with a SD of 1.00, slightly below the midpoint of the scale (midpoint=3.5). The mean baseline GAS score for boys was 3.01 (SD 1) while for girls it was 2.70 (SD 0.97), signifying significantly lower gaming disorder symptom severity in girls versus boys (*P*<.001). Regarding self-control mindset, the mean score at baseline was 3.26 (SD 1.01), which indicates the mindsets among students overall were average, with neither overly fixed nor growth mindsets prevailing. Furthermore, the mean baseline self-control mindset score for boys was 3.22 (SD 1.06) and for girls was 3.30 (SD 0.96), indicating a more fixed mindset at baseline in girls than in boys (*P*=.02).

The descriptive statistics and correlations between the variables across the 2 waves are presented in Table 2. All the variables were significantly correlated in the expected directions. The CLPM for GAS and self-control mindset fit the data (*χ*^*2*^_223_=1822.0, *P*<.001; CFI =0.94; RMSEA=0.047, 90% CI 0.045 to 0.049; SRMR=0.071). The estimates in the CLPM are summarized in Table 3. The results indicated GAS and self-control mindset were significantly related in both T1 and T2. Both stability paths were also statistically significant, and the 2 cross-lagged paths were significant as well. Self-control mindset predicted GAS over time, and GAS also prospectively predicted self-control mindset. For the CLPM analyses between self-control mindset and GT (Table 4; fit measures: *χ*^*2*^_37_=225.3, *P*<.001; CFI=0.98; RMSEA=0.039, 90% CI 0.035 to 0.045; SRMR=0.054), and between self-control mindset and self-control (Table 5; fit measures: *χ*^*2*^_317_=2740.7, *P*<.001; CFI=0.91; RMSEA=0.048, 90% CI 0.047 to 0.050; SRMR=0.081), it was found that GT and self-control predicted self-control mindset after 1 year, but self-control mindset did not predict them 1 year later.

**Table 2.** Descriptive statistics and correlation analysis of the variables. All correlations were significant at the *P*<.001 level.

	Mean (SD)	Self-control^a^ T1^b^	MS^c^ T1	GAS^d^ T1	GT^e^ T1	Self-control T2^f^	MS T2	GAS T2	GT T2
Self-control T1	3.47 (0.81)	—^g^	−0.47	−0.52	−0.14	0.68	−0.34	−0.39	−0.087
MS T1	3.26 (1.01)	−0.47	—	0.33	0.10	−0.33	0.44	0.28	0.088
GAS T1	2.85 (1)	−0.52	0.33	—	0.43	−0.41	0.26	0.62	0.32
GT T1	1.95 (1.77)	−0.14	0.10	0.43	—	−0.11	0.12	0.31	0.58
Self-control T2	3.32 (0.79)	0.68	−0.33	−0.41	−0.11	—	−0.45	−0.52	−0.11
MS T2	3.27 (0.98)	−0.34	0.44	0.26	0.12	−0.45	—	0.35	0.13
GAS T2	2.85 (0.98)	−0.39	0.28	0.62	0.31	−0.52	0.35	—	0.41
GT T2	1.82 (1.66)	−0.087	0.088	0.32	0.58	−0.11	0.13	0.41	—

a Range: 1-6; a higher value represents higher self-control.

b T1: time 1.

c MS: mindset regarding self-control (range: 1-6; a higher value represents a more fixed mindset).

d GAS: Game Addiction Scale (range: 1-6; a higher value represents more severe gaming disorder symptoms).

e GT: gaming time (range: 0-6; 0=none, 0.5=half hour, 1=1 hour, 2=2 hours, 3=3 hours, 4=4 hours, 5=5 hours, and 6=more than 5 hours; a higher value represents more average time spent playing video games each day).

f T2: time 2.

g Not applicable.

**Table 3.** Summary of the CLPM^a^ results between self-control mindsets and gaming disorder among the whole sample (N=3264).

	Covariance or coefficient (95% CI)	*P* value	Correlation or standardized coefficient
**Concurrent paths** ^**b**^			
MS^c^ T1^d^ and GAS^e^ T1	0.36 (0.32-0.4)	<.001	0.36
MS T2^f^ and GAS T2	0.38 (0.31-0.44)	<.001	0.38
**Autoregressive paths** ^**g**^			
MS T1 to MS T2	0.46 (0.4-0.51)	<.001	0.41
GAS T1 to GAS T2	0.62 (0.57-0.67)	<.001	0.52
**Cross-lagged paths** ^**g**^			
MS T1 to GAS T2	0.070 (0.02-0.12)	.006	0.059
GAS T1 to MS T2	0.11 (0.06-0.16)	<.001	0.099

a CLPM: cross-lagged panel model.

b The values represent the covariance or correlation coefficients.

c MS: mindset regarding self-control.

d T1: time 1.

e GAS: Game Addiction Scale.

f T2: time 2.

g The values represent the regression coefficients or standardized regression coefficients.

**Table 4.** Summary of the CLPM^a^ results between self-control mindsets and gaming time in the whole sample (N=3264).

	Covariance or coefficient (95% CI)	*P* value	Correlation or standardized coefficient
**Concurrent paths** ^b^			
MS^c^ T1^d^ and GT^e^ T1	0.2 (0.14 to 0.27)	<.001	0.12
MS T2^f^ and GT T2	0.12 (0.048 to 0.2)	.001	0.091
**Autoregressive paths** ^g^			
MS T1 to MS T2	0.5 (0.45 to 0.55)	<.001	0.44
GT T1 to GT T2	0.56 (0.52 to 0.6)	<.001	0.59
**Cross-lagged paths** ^g^			
MS T1 to GT T2	0.059 (−0.005 to 0.12)	.07	0.035
GT T1 to MS T2	0.021 (0.002 to 0.04)	.03	0.033

a CLPM: cross-lagged panel model.

b The values represent the covariance or correlation coefficients.

c MS: mindset regarding self-control.

d T1: time 1.

e GT: gaming time.

f T2: time 2.

g The values represent the regression coefficients or standardized regression coefficients.

**Table 5.** Summary of the CLPM^a^ results between self-control and self-control mindsets among the whole sample (N=3264).

	Covariance or coefficient (95% CI)	*P* value	Correlation or standardized coefficient
**Concurrent paths** ^b^			
MS^c^ T1^d^ and self-control T1	−0.52 (−0.55 to −0.48)	<.001	−0.52
MS T2^e^ and self-control T2	−0.54 (−0.59 to −0.48)	<.001	−0.54
**Autoregressive paths** ^f^			
MS T1 to MS T2	0.42 (0.36 to 0.48)	<.001	0.38
Self-control T1 to self-control T2	0.66 (0.61 to 0.71)	<.001	0.55
**Cross-lagged paths** ^f^			
MS T1 to self-control T2	−0.013 (−0.063 to 0.038)	.63	−0.01
Self-control T1 to MS T2	−0.15 (−0.21 to −0.089)	<.001	−0.13

a CLPM: cross-lagged panel model.

b The values represent the covariance or correlation coefficients.

c MS: mindset regarding self-control.

d T1: time 1.

e T2: time 2.

f The values represent the regression coefficients or standardized regression coefficients.

**Figure 1.**
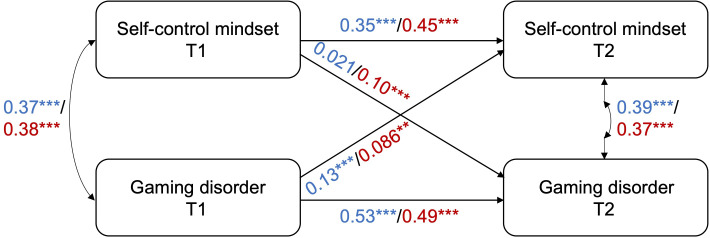
CLPM results between self-control mindset and gaming disorder for boys and girls separately. The values along the lines are the standardized coefficients of the relevant paths for boys (in blue) and girls (in red). *P≤.05, **P≤.01, ***P≤.001. Exact *P* values are reported in Tables S2 and S3 in **Multimedia Appendix 1**. CLPM: cross-lagged panel model; T: time.

**Figure 2.**
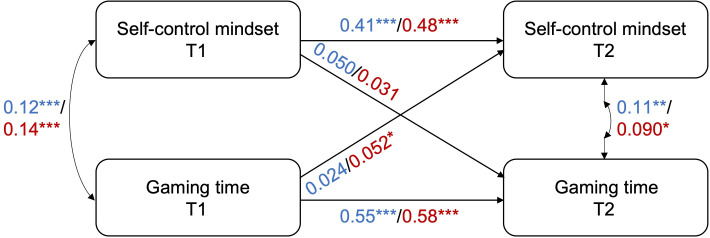
CLPM results between self-control mindset and gaming time for boys and girls separately. The values along the lines are the standardized coefficients of the relevant paths for boys (in blue) and girls (in red). *P≤.05, **P≤.01, ***P≤.001. Exact *P* values are reported in Tables S4 and S5 in **Multimedia Appendix 1**. CLPM: cross-lagged panel model; T: time.

**Figure 3.**
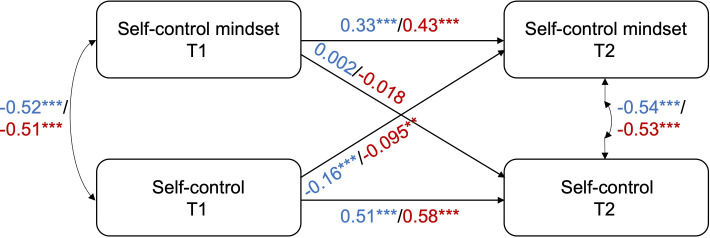
CLPM results between self-control mindset and self-control for boys and girls separately. The values along the lines are the standardized coefficients of the relevant paths for boys (in blue) and girls (in red). *P≤.05, **P≤.01, ***P≤.001. Exact *P* values are reported in Tables S6 and S7 in **Multimedia Appendix 1**. CLPM: cross-lagged panel model, T: time.

Subgroup CLPM analyses for girls and boys all had adequate fit as well (Table S1 in Multimedia Appendix 1). The CLPM results between self-control mindset and gaming disorder indicated that both the cross-lagged paths were significant in girl students, however, for boys, only the cross-lagged path from GAS in T1 to self-control mindset in T2 was significant, while the opposite was not statistically significant (Figure 1 and Tables S2 and S3 in Multimedia Appendix 1), signifying distinct patterns for the 2 genders. The CLPM results between self-control mindset and GT showed that only the cross-lagged path from GT at T1 to self-control mindset at T2 in girls was significant (Figure 2 and Tables S4 and S5 in Multimedia Appendix 1). For the cross-lagged effects between self-control mindset and self-control, self-control at T1 predicted self-control mindset at T2 in both boys and girls, while self-control mindset at T1 did not predict self-control at T2 (Figure 3 and Tables S6 and S7 in Multimedia Appendix 1).

## Discussion

### Principal Findings

Gaming disorder is a growing global concern. It is essential to identify the modifiable psychosocial factors influencing gaming disorder to develop suitable prevention strategies. The reciprocal associations between self-control mindsets and gaming disorder were examined with the current longitudinal study among children and adolescents in Hong Kong. The initial evidence of the directions of the aforementioned longitudinal associations helps identify self-control mindsets as a potential key psychological factor for gaming disorder prevention. The findings revealed substantive concurrent associations between fixed mindsets regarding self-control and severe gaming disorder symptoms at both assessment points. Moreover, the 1-year longitudinal association between fixed mindsets regarding self-control and gaming disorder has uncovered the long-term impact of self-control mindsets on gaming disorder and that gaming disorder could diminish one’s belief regarding the changeability of self-control.

The separate examinations among the 2 genders further unveiled the gender differences. The significant longitudinal reciprocal relationship only exists among girls. However, among boys, the gaming disorder symptom severity prospectively predicts fixed mindsets regarding self-control, but not reversely, which means boys’ gaming disorder symptoms predict their belief of whether they can improve their self-control.

To our knowledge, this is the first study on the reciprocal relationships between implicit theories and gaming disorder over time, and it provides important insights into this field of research. It is also a relatively large-scale study that is school-based and contains representative samples, adding to the applicability and generalizability of the findings.

### Comparison to Prior Work

Our study addresses a research gap by clarifying the directional links between gaming disorder and implicit theories of self-control. This study’s results revealed the bidirectional relationships between them. Regarding the prediction of self-control mindsets to gaming disorder symptoms after 1 year, our findings were consistent with the results of previous mindset intervention studies. For instance, a brief growth mindset intervention helped to reduce the incidence of clinically significant depressive symptoms during a 9-month follow-up, while depressive symptoms increased among adolescents with a fixed mindset of personality in the control condition [37]. In another study, a brief growth mindset of intelligence intervention was shown to improve secondary school students’ academic achievement and increase their enrollment in advanced courses [38]. This emerging body of research exhibits the effects of growth mindsets on mental health and performance-related outcomes. On the other hand, the findings regarding the alternative pathway of gaming disorder symptoms in predicting later self-control mindsets accorded with past findings that the baseline psychopathology of youth predicted increased fixed mindsets over time [23,30]. Synthesizing the CLPM results of this study, apart from gaming disorder symptoms, GT (especially in girls) and self-control levels also predicted later self-control mindsets, which denotes that mindsets are affected by various factors. The findings of this study demonstrated that experiences like addictive behaviors shape youths’ fixed mindsets. Like the findings supporting growth mindsets of intelligence are both antecedents and outcomes of greater academic achievement in youths [39], the bidirectional links between gaming disorder symptoms and implicit theories of self-control identified in our study underscore the reciprocal influences of these factors.

The association between self-control mindsets and gaming behaviors may be explained by the extant literature on how implicit theories impact self-regulation process [21,22]. First, it is postulated that growth mindsets regarding self-control could lead to reduced gaming disorder symptoms through self-reinforcing cycles of motivation and dedicated efforts to try new strategies [38]. Though self-control has been shown to have no causal relationships with gaming disorder by our recent study [40], individual factors encompassing self-control dimensions and gaming motivation explained substantive variance in gaming disorder, much more than gaming-related factors including game genre and platforms [41]. Second, the predictive path of self-control mindsets to gaming disorder symptoms was evident in girls but not in boys in the subgroup analyses, signifying that girls’ mindsets shall be more related to gaming behavior regulation. This finding aligned with past research findings regarding gender differences in gaming motives [14,42]. As boys score higher on all gaming motives except escape [14], strong gaming motives might undermine the possible effects of a growth mindset regarding self-control in preventing gaming disorder among boys. The gender differences found in this study also highlight the importance of gender in gaming [26] and more studies are needed to clarify the distinct gaming patterns among the 2 genders.

### Implications

The findings of this study have significant practical implications. First, a self-control mindset is a modifiable factor that can be used for developing gaming disorder prevention and early intervention. Given the strong concurrent and longitudinal association between self-control mindsets and gaming disorder revealed in this study, interventions that instill the belief that their self-control can become stronger may be potent to help prevent gaming disorder. Second, childhood and adolescence are ideal developmental stages for developing healthy self-regulatory digital product use. A growth mindset regarding self-control helps strengthen youths’ self-efficacy in self-control, especially for girls, and it is promisingly helpful to prevent students from developing gaming disorder in the future. Last but not least, interventions addressing self-control mindsets could be used as motivational interviewing and could be added to existing intervention programs or therapies for gaming disorder to boost patients’ confidence and motivation to regain control over their gaming behaviors. A top-up session addressing self-control mindsets will be likely to amplify the efficacy of the original interventions.

### Limitations and Future Directions

There are limitations to this study. First, causality cannot be inferred from this study. Self-control mindsets and gaming disorder could still be causally unrelated, and their association could be accounted for by other variables. A recent study revealed a unidirectional negative relationship from self-control to gaming disorder via traditional CLPM analysis but no prospective relationship in a random intercept CLPM analysis [40]. Longitudinal studies containing more waves of data with applying more rigorous analysis methods and studies with experimental designs are needed to inform us better of the causality between self-control mindsets and gaming disorder. Second, though this study’s main aim was not the underlying mediators in the relationship between mindsets of self-control and gaming disorder but the directional relationship between them, longitudinal mediation models need to be tested in future research to figure out the relationships between mindsets, self-control, and gaming disorder. Third, the measures including participants’ self-control mindsets and gaming disorder symptoms were self-reported; as such, there may be subjective biases influencing the objectivity of this study’s findings, although we used attention-checking questions to ensure the quality of the survey. Moreover, GT cannot be perfectly assessed by asking participants to indicate their average GT each day using a scale with several discrete durations; rather, screen monitor tools should be used to record exact GT in future research. Fourth, this study’s sample was just Hong Kong children and adolescents and thus the findings might not be able to generalize to a wider population. Future research should validate our study findings with larger and more representative samples.

### Conclusion

Internet gaming plays an important role in leisure and educational activities in the digital age. This study is a pioneering investigation into the reciprocal relationships between implicit theories and gaming disorder. This study’s results highlight that gaming disorder was associated with a more fixed mindset about self-control after one year in both genders, while only girls’ fixed mindsets regarding self-control significantly led to subsequent gaming disorder symptoms after 1 year. The findings lay the groundwork for future research and practices for cultivating growth mindsets to prevent adolescents from developing gaming disorder.

## Supplementary material

10.2196/59441 Multimedia Appendix 1 Supplementary tables.

## Abbreviations

CFI: comparative fit index
CLPM: cross-lagged panel model
*DSM-5*: *Diagnostic and Statistical Manual of Mental Disorders, Fifth Edition*
GAS: Game Addiction Scale
GT: gaming time
*ICD-11*: *International Classification of Diseases, Eleventh Edition*
IGD: internet gaming disorder
RMSEA: root mean square error of approximation
SRMR: standardized root mean square residual
