# Supplementary material for: Reciprocal Relationship Between Self-Control Belief and Gaming Disorder in Children and Adolescents: Longitudinal Survey Study
Source: JMIR Serious Games. 2025 Jan 15;13:e59441. doi: 10.2196/59441 (PMC11769689; doi:10.2196/59441)
Supplement: Multimedia Appendix 1 [file games-v13-e59441-s001.docx]

*Socioeconomic status (SES)* was measured using the adapted Family Affluence Scale [1]. Four items were applied to assess participants’ family affluence level: car ownership (0: No; 1: Yes), the number of household appliances (television, refrigerator, microwave oven, washing machine, dishwasher/cutlery washing and sanitizing machine, vacuum cleaner/ robotic vacuum, rice cooker and air conditioner; 1: 1-4 pieces; 2: 5-8 pieces), the number of learning devices (desktop computer, iPad or tablets, notebook computer, smartphone, and e-book reader; 1: 1-2 pieces; 2: 3-5 pieces), and household Internet connection satisfaction (0: totally unsatisfied, partially unsatisfied or not sure; 1: basically satisfied or totally satisfied). The scores on the 4 items were summed to get a total score (range: 2-6), with higher scores representing more family affluence. Finally, participants were classified into three levels of SES: low (total score = 2), medium (total score = 3-5), and high (total score = 6).

**References**

1. Currie, C.E., et al., *Indicators of socioeconomic status for adolescents: the WHO Health Behaviour in School-aged Children Survey.* Health Education Research, 1997. **12**(3): p. 385-397.

**Table S1.** Fit measures of subgroup CLPM analyses.

| CLPM | sex | χ^2^ | *df* | CFI | RMSEA (90% CI) | SRMR |
| --- | --- | --- | --- | --- | --- | --- |
| MS---GAS | boys | 969.82 | 223 | .94 | .047 [.044, .050] | .071 |
|  | girls | 1107.90 | 223 | .94 | .048 [.045, .051] | .072 |
| MS---GT | boys | 131.42 | 37 | .98 | .041 [.033, .048] | .054 |
|  | girls | 141.17 | 37 | .98 | .040 [.033, .048] | .054 |
| MS---Self-control | boys | 1515.62 | 317 | .91 | .050 [.047, .052] | .085 |
|  | girls | 1542.10 | 317 | .92 | .047 [.045, .050] | .080 |

*Note:* CLPM: cross-lagged panel model; MS: mindset regarding self-control; GAS: game addiction scale; GT: gaming time; CFI: comparative fit index; RMSEA: root-mean-square error of approximation; CI: confidence interval; SRMR: standardized root-mean-square residual.

**Table S2.** Summary of the CLPM results between self-control mindsets and gaming disorder among boys.

| Boys (*N* = 1539) | Covariance/Coefficient | *P* | Correlation/Standardized coefficient |
| --- | --- | --- | --- |
| Concurrent paths ^a^ |  |  |  |
| MS T1 and GAS T1 | **.37 [.32, .43]** | **< .001** | **.37** |
| MS T2 and GAS T2 | **.39 [.30, .48]** | **< .001** | **.39** |
| Autoregressive paths ^b^ |  |  |  |
| MS T1 to MS T2 | **.39 [.30, .47]** | **< .001** | **.35** |
| GAS T1 to GAS T2 | **.64 [.56, .71]** | **< .001** | **.53** |
| Cross-lagged paths ^b^ |  |  |  |
| MS T1 to GAS T2 | .025 [-.050, .10] | .51 | .021 |
| GAS T1 to MS T2 | **.15 [.069, .23]** | **< .001** | **.13** |

*Note:* CLPM: cross-lagged panel model; MS: mindset regarding self-control; GAS: game addiction scale; T1: Time 1; T2: Time 2. 95% Confidence Intervals are given in brackets in the Covariance/Coefficient column. Bold values represent significance at *P* < .05.

^a^ The values represent the covariance/correlation coefficients.

^b^ The values represent the regression coefficients/standardized regression coefficients.

**Table S3.** Summary of the CLPM results between self-control mindsets and gaming disorder among girls.

| Girls (*N* = 1725) | Covariance/Coefficient | *P* | Correlation/Standardized coefficient |
| --- | --- | --- | --- |
| Concurrent paths ^a^ |  |  |  |
| MS T1 and GAS T1 | **.38 [.33, .43]** | **< .001** | **.38** |
| MS T2 and GAS T2 | **.37 [.28, .46]** | **< .001** | **.37** |
| Autoregressive paths ^b^ |  |  |  |
| MS T1 to MS T2 | **.51 [.44, .58]** | **< .001** | **.45** |
| GAS T1 to GAS T2 | **.58 [.52, .65]** | **< .001** | **.49** |
| Cross-lagged paths ^b^ |  |  |  |
| MS T1 to GAS T2 | **.12 [.053, .19]** | **.001** | **.10** |
| GAS T1 to MS T2 | **.098 [.031, .17]** | **.004** | **.086** |

*Note:* CLPM: cross-lagged panel model; MS: mindset regarding self-control; GAS: game addiction scale; T1: Time 1; T2: Time 2. 95% Confidence Intervals are given in brackets in the Covariance/Coefficient column. Bold values represent significance at *P* < .05.

^a^ The values represent the covariance/correlation coefficients.

^b^ The values represent the regression coefficients/standardized regression coefficients.

**Table S4**. Summary of the CLPM results between self-control mindsets and gaming time among boys.

| Boys (*N* = 1539) | Covariance/Coefficient | *P* | Correlation/Standardized coefficient |
| --- | --- | --- | --- |
| Concurrent paths ^a^ |  |  |  |
| MS T1 and GT T1 | **.22 [.12, .32]** | **< .001** | **.12** |
| MS T2 and GT T2 | **.15 [.037, .27]** | **.01** | **.11** |
| Autoregressive paths ^b^ |  |  |  |
| MS T1 to MS T2 | **.45 [.37, .52]** | **< .001** | **.41** |
| GT T1 to GT T2 | **.51 [.45, .58]** | **< .001** | **.55** |
| Cross-lagged paths ^b^ |  |  |  |
| MS T1 to GT T2 | .086 [-.019, .19] | .11 | .050 |
| GT T1 to MS T2 | .015 [-.010, .039] | .24 | .024 |

*Note:* CLPM: cross-lagged panel model; MS: mindset regarding self-control; GT: gaming time; T1: Time 1; T2: Time 2. 95% Confidence Intervals are given in brackets in the Covariance/Coefficient column. Bold values represent significance at *P* < .05.

^a^ The values represent the covariance/correlation coefficients.

^b^ The values represent the regression coefficients/standardized regression coefficients.

**Table S5**. Summary of the CLPM results between self-control mindsets and gaming time among girls.

| Girls (*N* = 1725) | Covariance/Coefficient | *P* | Correlation/Standardized coefficient |
| --- | --- | --- | --- |
| Concurrent paths ^a^ |  |  |  |
| MS T1 and GT T1 | **.23 [.14, .32]** | **< .001** | **.14** |
| MS T2 and GT T2 | **.11 [.022, .20]** | **.02** | **.090** |
| Autoregressive paths ^b^ |  |  |  |
| MS T1 to MS T2 | **.54 [.48, .61]** | **< .001** | **.48** |
| GT T1 to GT T2 | **.55 [.48, .61]** | **< .001** | **.58** |
| Cross-lagged paths ^b^ |  |  |  |
| MS T1 to GT T2 | .048 [-.028, .12] | .22 | .031 |
| GT T1 to MS T2 | **.037 [.006, .068]** | **.02** | **.052** |

*Note:* CLPM: cross-lagged panel model; MS: mindset regarding self-control; GT: gaming time; T1: Time 1; T2: Time 2. 95% Confidence Intervals are given in brackets in the Covariance/Coefficient column. Bold values represent significance at *P* < .05.

^a^ The values represent the covariance/correlation coefficients.

^b^ The values represent the regression coefficients/standardized regression coefficients.

**Table S6**. Summary of the CLPM results between self-control and self-control mindsets among boys.

| Boys (*N* = 1539) | Covariance/Coefficient | *P* | Correlation/Standardized coefficient |
| --- | --- | --- | --- |
| Concurrent paths ^a^ |  |  |  |
| MS T1 and self-control T1 | **-.52 [-.57, -.46]** | **< .001** | **-.52** |
| MS T2 and self-control T2 | **-.54 [-.63, -.46]** | **< .001** | **-.54** |
| Autoregressive paths ^b^ |  |  |  |
| MS T1 to MS T2 | **.36 [.27, .45]** | **< .001** | **.33** |
| Self-control T1 to self-control T2 | **.59 [.51, .67]** | **< .001** | **.51** |
| Cross-lagged paths ^b^ |  |  |  |
| MS T1 to self-control T2 | .002 [-.075, .079] | .96 | .002 |
| Self-control T1 to MS T2 | **-.17 [-.26, -.087]** | **< .001** | **-.16** |

*Note:* CLPM: cross-lagged panel model; MS: mindset regarding self-control; T1: Time 1; T2: Time 2. 95% Confidence Intervals are given in brackets in the Covariance/Coefficient column. Bold values represent significance at *P* < .05.

^a^ The values represent the covariance/correlation coefficients.

^b^ The values represent the regression coefficients/standardized regression coefficients.

**Table S7**. Summary of the CLPM results between self-control and self-control mindsets among girls.

| Girls (*N* = 1725) | Covariance/Coefficient | *P* | Correlation/Standardized coefficient |
| --- | --- | --- | --- |
| Concurrent paths ^a^ |  |  |  |
| MS T1 and self-control T1 | **-.51 [-.57, -.46]** | **< .001** | **-.51** |
| MS T2 and self-control T2 | **-.53 [-.61, -.46]** | **< .001** | **-.53** |
| Autoregressive paths ^b^ |  |  |  |
| MS T1 to MS T2 | **.49 [.41, .57]** | **< .001** | **.43** |
| Self-control T1 to self-control T2 | **.72 [.65, .78]** | **< .001** | **.58** |
| Cross-lagged paths ^b^ |  |  |  |
| MS T1 to self-control T2 | -.023 [-.089, .043] | .50 | -.018 |
| Self-control T1 to MS T2 | **-.11 [-.18, -.035]** | **.004** | **-.095** |

*Note:* CLPM: cross-lagged panel model; MS: mindset regarding self-control; T1: Time 1; T2: Time 2. 95% Confidence Intervals are given in brackets in the Covariance/Coefficient column. Bold values represent significance at *P* < .05.

^a^ The values represent the covariance/correlation coefficients.

^b^ The values represent the regression coefficients/standardized regression coefficients.

Questionnaires used in this study:

1) Gaming time:

**A^2^** On average per day, how many hours do you spend on the following activities: (more than 30 minutes counts as 1 hour):

|  | | None | Half Hour | One  Hour | Two  Hours | Three  Hours | Four  Hours | Five  Hours | More |
| --- | --- | --- | --- | --- | --- | --- | --- | --- | --- |
| f | Playing video games | □ | □ | □ | □ | □ | □ | □ | □ |

2) Gaming disorder symptoms:

| **# A^9^** | | Strongly  disagree | Disagree | Slightly  disagree | Slightly  agree | Agree | Strongly  agree |
| --- | --- | --- | --- | --- | --- | --- | --- |
| a | I have thought all day long about playing a game. | □ | □ | □ | □ | □ | □ |
| b | I have played longer than intended. | □ | □ | □ | □ | □ | □ |
| c | I have played games and missed planned work. | □ | □ | □ | □ | □ | □ |
| d | Others have unsuccessfully tried to reduce my time spent on games. | □ | □ | □ | □ | □ | □ |
| e | I have felt upset when I was unable to play. | □ | □ | □ | □ | □ | □ |
| f | I have had arguments with others (eg, family and friends) over my time spent on games. | □ | □ | □ | □ | □ | □ |
| g | I have neglected important activities (eg, school, work, and sports) to play games. | □ | □ | □ | □ | □ | □ |

3) Self-control:

| **# C^1^** | | Strongly  disagree | Disagree | Slightly  disagree | Slightly  agree | Agree | Strongly  agree |
| --- | --- | --- | --- | --- | --- | --- | --- |
| a | I am good at resisting temptation. | □ | □ | □ | □ | □ | □ |
| b | I have a hard time breaking bad habits. | □ | □ | □ | □ | □ | □ |
| c | I am lazy. | □ | □ | □ | □ | □ | □ |
| d | I do certain things that are bad for me, if they are fun. | □ | □ | □ | □ | □ | □ |
| e | Pleasure and fun sometimes keep me from getting work done. | □ | □ | □ | □ | □ | □ |
| f | I have trouble concentrating. | □ | □ | □ | □ | □ | □ |
| g | I am able to work effectively toward long-term goals. | □ | □ | □ | □ | □ | □ |
| h | Sometimes I can’t stop myself from doing something, even if I know it is wrong. | □ | □ | □ | □ | □ | □ |
| i | I often act without thinking through all the alternatives. | □ | □ | □ | □ | □ | □ |

4) Self-control mindset:

| **# C^2^** | | Strongly  disagree | Disagree | Slightly  disagree | Slightly  agree | Agree | Strongly  agree |
| --- | --- | --- | --- | --- | --- | --- | --- |
| a | People have a certain amount of self-control, there isn’t much they can do to change that. | □ | □ | □ | □ | □ | □ |
| b | People cannot substantially develop or change their self-control. | □ | □ | □ | □ | □ | □ |
| c | To be honest, you can’t really change your self-control. | □ | □ | □ | □ | □ | □ |
| d | No matter what people do, their self-control cannot be substantially changed or developed. | □ | □ | □ | □ | □ | □ |
